# Harmful effects from one puff of shisha-pen vapor: methodological and interpretational problems in the risk assessment analysis

**DOI:** 10.1186/s12971-016-0086-7

**Published:** 2016-07-07

**Authors:** Konstantinos E. Farsalinos, Frank Baeyens

**Affiliations:** Department of Cardiology, Onassis Cardiac Surgery Center, Sygrou 356, Kallithea, 17674 Greece; Department of Pharmacy, University of Patras, Rio, 26100 Greece; Faculty of Psychology and Educational Sciences, KU Leuven-University of Leuven, Tiensestraat 102, 3000 Leuven, Belgium

**Keywords:** Electronic cigarettes, Propylene glycol, Glycerol, Margin of exposure

## Abstract

With this letter we express our concerns about the applicability of the proposed Margin of Exposure analysis as a method of risk assessment for propylene glycol and glycerol exposure from a shisha-pen type electronic cigarette. The studies used to determine the Margin of Exposure were evaluating the effects in humans or animals of continuous exposure to these chemicals in every single breath, whereas electronic cigarettes are used intermittently by consumers, resulting in lower and discontinuous exposure. Moreover, the authors make no clear distinction between irritation and harm, neither do they discuss the effects of exposure compared to continuous smoking.

We read with particular interest the study by Kienhuis et al. [1] concerning the potentially harmful effects of propylene glycol and glycerol exposure from a shisha-pen type electronic cigarette. With this letter we express our concerns that the title and conclusions of the study may not be supported by the presented evidence.

The toxicological Point of Departure (PoD) for the Margin of Exposure (MoE) calculations for propylene glycol was derived from a human study evaluating aerosol mist exposure during an aviation emergency training exercise [2]. Firstly, it is unclear if the authors used industrial grade (which is commonly used in aviation for de-icing) instead of pharmaceutical grade propylene glycol (which is used in electronic cigarettes). Moreover, the main effects observed were - relatively minor - ocular and throat irritation (pre-post exposure increases from 5 to 14 for ocular irritation and from 7 to 20 for throat irritation on a 100 mm visual analogue rating scale). Obviously, ocular irritation is associated with environmental exposure and not inhalation. Throat irritation is commonly called “throat hit” by smokers or vapers, and is in fact a desired effect. Propylene glycol is probably contributing to this by its humectant effect, and this should not be defined as a harmful effect. Additionally, the effects were observed after continuous exposure for 1 min, which means that the participants were exposed to those levels for 12 consecutive breaths (assuming resting breathing rate). It is highly unlikely for consumers to use electronic cigarettes at that rate, considering the puffing patterns in experienced users after abstaining for 8 h [3].

For glycerol, Kienhuis et al. referred to data from animal exposure studies in which animals were continuously exposed to glycerol for 6 h per day, meaning that they were exposed to the chemicals in every single breath during the exposure period. An electronic cigarette use topography study showed that, during a 5 min session, the average inhalation time was approximately 1 s and the average interpuff interval was 23 s [3]. This means that the levels of propylene glycol and glycerol in the lungs will be significantly reduced between puffs, and would become essentially zero during the inter-session intervals. Therefore, the use of continuous exposure studies for risk assessment (or as PoD in a MoE analysis) of propylene glycol or glycerol exposure from electronic cigarette use is inappropriate and probably over-estimates any risk. Such an approach would be useful when examining systemic effects, by evaluating total absorption, but is unlikely to be of value when examining local effects considering the important differences in exposure patterns.

Additionally, we detected an inaccurate statement, mentioning that: “*It is not clear if irreversible effects will occur after prolonged use but an animal study showed that repeated exposure (6 h per day; 5 days per week) for 90 days at 1000 and 2200 mg/m*^*3*^*caused irreversible respiratory damage*” and citing a study by Suber et al. [4]. It is unclear if Kienhuis et al. refer to the elevation in the number of goblet cells or elevated mucin production by pre-existing goblet cells in the nasal turbinates, which is probably related to the dehydrating effects of propylene glycol and was not considered as an indication of irreversible damage. In fact, Suber et al. clearly mention that: “*The changes observed in organ weights and clinical pathology parameters did not indicate a toxic effect on any single organ system or blood component*” and concluded that their study “*confirms that propylene glycol would not cause adverse health effects when exposures are based upon these no-observed-effect levels*”. Similar conclusions were reported in other animal studies [5, 6].

Last but not least, the authors discuss harmful effects without mentioning how these effects would be compared to smoking. Since electronic cigarettes are mainly used as an alternative to smoking, it would be important to address any concerns in the context of continuous smoking. For example, in a recent clinical trial by Adriaens et al., smokers who (partially or completely) switched to electronic cigarettes were found to occasionally report some complaints including dry/irritated mouth and throat, but the overall level of subjective complaints was significantly *lower* in users of electronic cigarettes than in a control group of smokers who continued to smoke regular cigarettes [7]. Considering all of the above, as well as the lack of a clear distinction between irritation and harm, we think that the evidence does not support the conclusion that there is potential for harmful effects after one puff of a shisha-pen type electronic cigarette. Of course, this does not necessarily mean that propylene glycol inhalation is absolutely harmless, but this cannot be addressed with the approach and methodology chosen by Kienhuis et al.

## Abbreviations

MoE, margin of exposure; PoD, point of departure
